# 3D-QSAR Studies of Dihydropyrazole and Dihydropyrrole Derivatives as Inhibitors of Human Mitotic Kinesin Eg5 Based on Molecular Docking

**DOI:** 10.3390/molecules17022015

**Published:** 2012-02-17

**Authors:** Xingyan Luo, Mao Shu, Yuanqiang Wang, Jin Liu, Wenjuan Yang, Zhihua Lin

**Affiliations:** School of Pharmacy and Bioengineering, Chongqing University of Technology, Chongqing 400054, China

**Keywords:** Eg5 inhibitors, LigandFit docking, 3D-QSAR

## Abstract

Human mitotic kinesin Eg5 plays an essential role in mitoses and is an interesting drug target against cancer. To find the correlation between Eg5 and its inhibitors, structure-based 3D-quantitative structure–activity relationship (QSAR) studies were performed on a series of dihydropyrazole and dihydropyrrole derivatives using comparative molecular field analysis (CoMFA) and comparative molecular similarity indices analysis (CoMSIA) methods. Based on the LigandFit docking results, predictive 3D-QSAR models were established, with cross-validated coefficient values (q^2^) up to 0.798 for CoMFA and 0.848 for CoMSIA, respectively. Furthermore, the CoMFA and CoMSIA models were mapped back to the binding sites of Eg5, which could provide a better understanding of vital interactions between the inhibitors and the kinase. Ligands binding in hydrophobic part of the inhibitor-binding pocket were found to be crucial for potent ligand binding and kinases selectivity. The analyses may be used to design more potent EG5 inhibitors and predict their activities prior to synthesis.

## 1. Introduction

The human mitotic kinesin Eg5 is one member of the Kinesin-5 subfamily, which function is helping the formation of bipolar mitotic spindle, and has been identified as a potential target for new drug development in cancer chemotherapy [1]. Many researches had been performed to discover new inhibition mechanismS of Eg5, such as RNA interference [2], potential inhibitors, like monoastral, which produces cells arrested in mitosis with a characteristic monoastral spindle phenotype [1,3,4,5,6,7,8,9]. As multidrug resistance (MDR) of anticancer drug like taxanes and vinca alkaloids has become a serious problem in cancer chemotherapy [10,11], the Eg5 inhibitors have been tested for their susceptibility to the PgP efflux pump and some of them have been validated for greater potential to overcome MDR [12]. Thus, Eg5 inhibitors have been discovered for potential anticancer drugs [13,14,15]. 

Merck Research Laboratories scientists have reported dihydropyrazole and dihydropyrrole inhibitors with inhibitory bioactivity against Eg5 in the low nanomolar IC_50_ range (from 1.2 nM to 829 nM) [16,17,18]. The compounds were used under the same conditions of an *in vitro* screening procedure based on the inhibition of the ATP kinase activity of Eg5, which like STLC leads to mitotic arrest by slowing ADP release from the catalytic site of Eg5 so that induces cancer cell death by the apoptotic pathway [19]. Some of these inhibitors showed good potency in Pgp-overexpressing cells. Thus dihydropyrazole and dihydropyrrole derivatives were described as Eg5 inhibitors that possess good to excellent intrinsic potency, aqueous solubility, low MDR ratios, limited hERG affinity, and excellent *in vivo* ability [18]. Meanwhile, Kaan *et al*. determined the crystal structure of the Eg5-STLC complex (PDB: 2WOG) [20], and reported that the inhibitive mechanism involved the fact that loop L5 of the final inhibitor-bound state was swung downwards to close the inhibitor-binding pocket, its helix α4 has rotated by approx 15° and the neck-linker has adopted a docked conformation. There have some articles that have adopted computer aided drug design to find new kinds of inhibitors of Eg5, but they just explored the structure-activity relationships (SAR), not the QSAR of Eg5-inhibitors [12,19,21,22,23]. In this paper, we examine the three dimensional quantitative structure-activity relationships (3D-QSAR) using comparative molecular field analysis (CoMFA), comparative molecular similarity indices analysis (CoMSIA) [24,25,26,27,28,29,30] and molecular docking (LigandFit Docking [31]) analyses, that provide insinhts into the relationship between the structural information of dihydropyrazole and dihydropyrrole inhibitors and their inhibitory potency, aimed at providing valuable guidance for the design of EG5 inhibitor compounds with highly anticancer activity.

## 2. Materials and Methods

### 2.1. Data Set

Thirty-seven dihydropyrazoles and dihydropyrroles derivatives were collected from Merck publications [16,17,18]. The biological data was represented as pIC_50_. It’s important to select training data and molecular alignment rules for building a good 3D-QSAR model [32] The structures of all compounds were download from the binding database [33]. The biological data was considered comparable and randomly divided into a training set (30 compounds) and a test set (seven compounds, mark with “*”), as shown in Table 1.

### 2.2. Molecular Docking

Molecular docking studies was performed using the LigandFit Docking module in the Receptor- Ligand Interaction package of the Accelrys Discovery Studio 2.5 software [31]. Atomic coordinates for the EG5 complex with STLC, used for docking modeling, have been deposited in the Protein DataBank (PDB code: 2WOG) [20], the original ligand was removed from the coordinated set. All chemical compounds and their possible poses were evaluated by scoring functions.

**Table 1:** The molecular structures and inhibitory activity of Eg5 inhibitors. 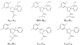

NO.	X	R\R_1_	R_2_	IC50 (nM)	pIC50
**A**_01_	F	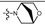	-	1.2	8.9208
**A**_02_ *	CF_3_	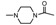	-	2.0	8.699
**A**_03_	F	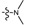	-	2.1	8.6778
**A**_04_	F	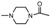	-	3.8	8.4202
**A**_05_ *	F	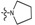	-	3.8	8.4202
**A**_06_	Cl	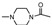	-	3.9	8.4089
**A**_07_	F	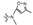	-	4.0	8.3979
**A**_08_	F	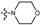	-	4.2	8.3768
**A**_09_	Br	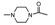	-	4.7	8.3279
**A**_10_ *	F	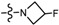	-	5.2	8.284
**A**_11_	CF_3_	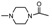	-	10.1	7.9957
**B**_01_	F	-(CH_2_)_4_-		26.0	7.585027
**B**_02_ *	F	-(CH_2_)_3_-		55.0	7.259637
**B**_03_	F	-(CH_2_)_5_-		85.0	7.070581
**B**_04_	F	-(CH_2_)_2_O(CH_2_)_2_-		122.0	6.91364
**B**_05_	F	-NHBn	-	100.0	7
**B**_06_	F	-NMe_2_	-	103.0	6.9872
**B**_07_	F	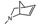	-	119.0	6.9245
**B**_08_ *	F	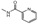	-	391.0	6.4078
**B**_09_	F	-NMe_2_O	-	585.0	6.2328
**B**_10_	F	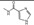	-	686.0	6.1637
**B**_11_	F	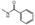	-	829.0	6.0814
**B**_12_	F	CH_2_ CH_3_	(CH_2_)_3_NH_2_	44.0	7.3565
**B**_13_	F	CH_2_ CH_3_	(CH_2_)_4_NH_2_	67.0	7.1739
**B**_14_	F	CH_3_	CH_3_	284.0	6.5467
**B**_15_	F	CH_2_ CH_3_	(CH_2_)_2_NH_2_	390.0	6.4089
**B**_16_ *	F	CH_2_ CH_3_	(CH_2_)_4_OH	697.0	6.1568
**B**_17_	F	CH_2_ CH_3_	(CH_2_)_3_OH	745.0	6.1278
**C**_01_	F	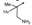	-	5.2	8.284
**C**_02_	F	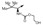	-	7.4	8.1308
**C**_03_ *	F	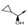	-	11.0	7.9586
**C**_04_	F	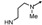	-	16.0	7.7959
**C**_05_	F	NMe_2_	-	38.0	7.4202
**C**_06_	F	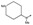	-	50.0	7.301
**C**_07_	F	NMe_2_	-	84.0	7.075721
**C**_08_	F	Me	-	94.0	7.026872
**C**_09_	F	t-Bu	-	113.0	6.946922

* Stands for molecules belonging to the test set (seven compounds).

### 2.3. 3D-QSAR Modeling *[25,26]*

3D-QSAR models were constructed by using CoMFA and CoMSIA in the SYBYL program package. Parameters of CoMFA and CoMSIA were the default values. The cut off value was set 30 kcal/mol. With standard options for scaling of variables, the regression analysis was performed using the “leave-one-out’’ cross-validation partial least squares method. The final non-cross-validated model was developed with a no validation PLS analysis.

## 3. Results and Discussion

### 3.1. Molecular Docking

**Figure 1:**
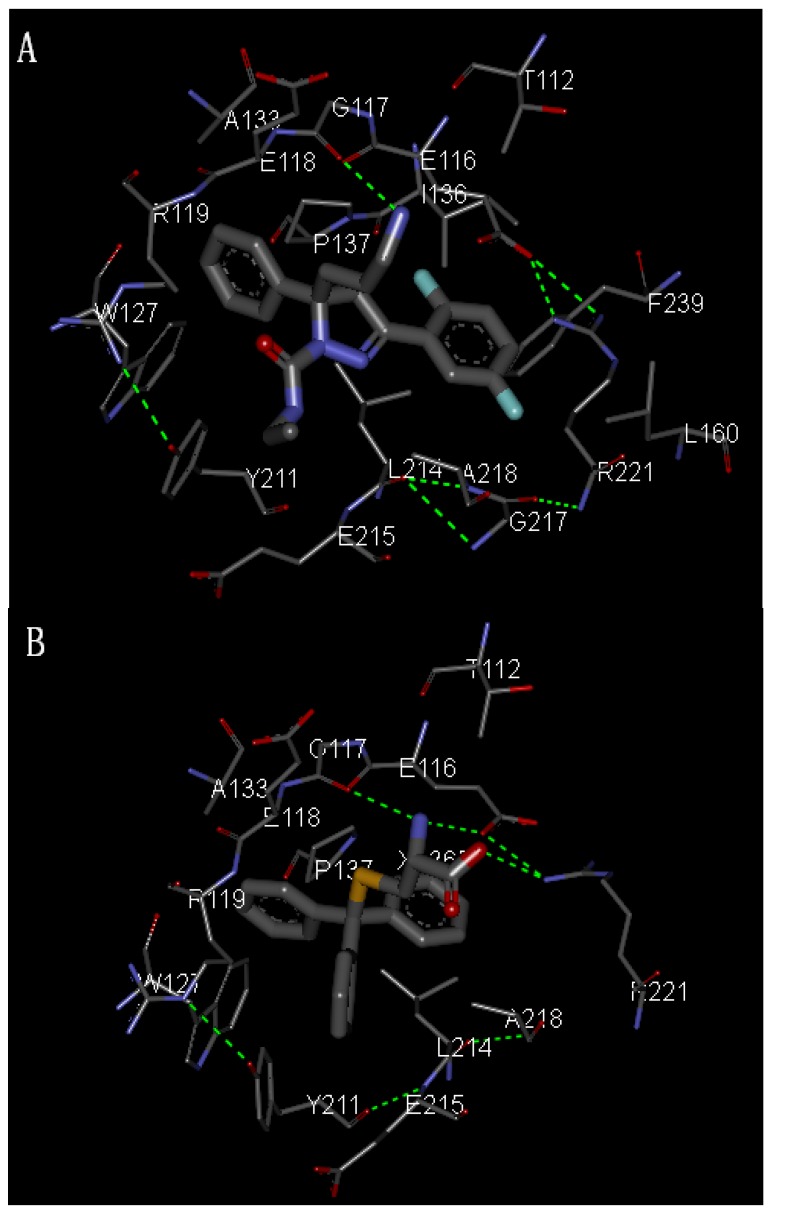
(**A**) Eg5 X-ray structure of the allosteric binding site with the B_12_ inhibitors in the binding site (PDB: 2G1Q); (**B**) Stereo plot showing STLC in the binding site (PDB: 2WOG).

The molecular modeling results using a molecular docking method revealed the possible molecular orientation of STLC and the derivatives in the binding pocket of Eg5 (Figure 1). STLC and B12 were buried in the pocket by the E116, E117, E118, R119 *etc*., and it was considered that they shared the same binding site. This suggests a similar binding mode for the dihydropyrazoles, dihydropyrroles and the *S*-trityl-L-cysteine (STLC), and those compounds can arrest cells in mitosis with a characteristic monoastral phenotype [12,20]. 

**Figure 2:**
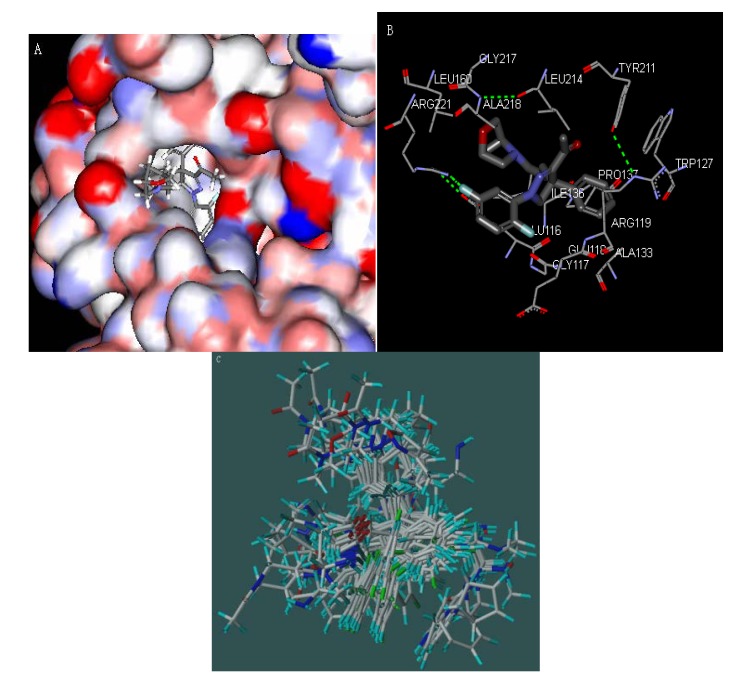
(**A**) Surface diagram showing the positions of the 1,4-diaryl-4,5-dihydropyrazole (A_01_) in the pocket (PDB code: 2WOG); (**B**) Stereo plot showing 1,4-diaryl-4,5-dihydropyrazole (A_01_) in the pocket (PDB code: 2WOG); (**C**) Overall of alignment of the 37 inhibitors obtained by the molecular docking.

Figure 2A,B show how the substituted 4, 5-dihydropyrazole derivate (A_01_) is buried in the binding pocket of Glu116, Gly117, and Glu118, while the R group is in the solvent-exposed region of the protein. Ligands binding in hydrophobic part of the inhibitor-binding pocket were found to be crucial for potent ligand binding and kinase selectivity. Further QSAR analysis was obtained by optimal conformation of inhibitors based on the LigandFit docking results. The optimal conformation of the 37 inhibitors is shown in the Figure 2C.

### 3.2. CoMFA and CoMSIA of 3D-QASR Models

The results of the CoMFA and CoMSIA methods are summarized in Table 2. In the CoMFA model the cross-validated coefficient q^2^ is 0.798, regression coefficient r^2^ is 0.980, and number of optimum components is 4, Standard Error of Estimate (SEE) is 0.127.

**Table 2:** Statistical parameters of CoMFA and CoMSIA models for the training set based on the molecular docking.

Model	q^2^	n	r^2^	SEE	F	S%	E%	H%	D%	A%
**CoMFA**	0.798	4	0.980	0.127	304.977	34.9	65.1			
**CoMSIA**	0.848	4	0.992	0.08	769.202	8.7	29.2	16.6	24.6	20.9

q^2^ is LOO cross-validated correlated coefficient, r^2^ is non-cross-validated coefficient. n is the optimal number of components in the non-cross-validated coefficient analysis. SE is Standard Error of Estimate. F is the ratio of r^2^ to 1.0- r^2^. S% stands for contribution of steric field. E% stands for contribution of electrostatic field. H% stands for contribution of hydrophobic field. D% stands for contribution of hydrogen-bond donor field. A% stands for hydrogen-bond acceptor field.

In the CoMSIA model, the q^2^ was as high as 0.848, r^2^ was 0.992, with the number of components = 4 and SEE was 0.08. In the test set, the regression coefficients r^2^ are 0.955, and 0.920, respectively, implying that the CoMFA and CoMSIA models were reliable and powerful in predicting pIC_50_ values. Table 3 shows the predicted pIC_50_ and residues between predicted and experimentally measured pIC_50_ values.

**Table 3:** Comparison of experimental activities (pIC50 values), predicted activities (pIC50 values) and residual values of all the 37 inhibitors were shown in the CoMFA and CoMSIA models.

No.	Actual pIC_50_	CoMFA		CoMSIA	
		Predicted	Residues	Predicted	Residues
**A**_01_	8.9208	8.999	−0.0777	8.954	−0.0337
**A**_02_ *	8.699	8.495	0.2036	8.17	0.5294
**A**_03_	8.6778	8.679	−0.0015	8.587	0.0909
**A**_04_	8.4202	8.339	0.0816	8.459	−0.0387
**A**_05_ *	8.4202	8.708	−0.2881	8.603	−0.1826
**A**_06_	8.4089	8.445	−0.0361	8.443	−0.0342
**A**_07_	8.3979	8.448	−0.0499	8.424	−0.0263
**A**_08_	8.3768	8.399	−0.0218	8.268	0.109
**A**_09_	8.3279	8.334	−0.0058	8.377	−0.0494
**A**_10_ *	8.284	8.54	−0.2558	8.454	−0.1702
**A**_11_	7.9957	7.977	0.0185	7.977	0.0188
**B**_01_	7.585	7.747	−0.1617	7.685	−0.0997
**B**_02_ *	7.2596	7.372	−0.1129	7.408	−0.1481
**B**_03_	7.0706	7.148	−0.0774	7.009	0.0617
**B**_04_	6.9136	6.761	0.1527	6.768	0.1461
**B**_05_	7	6.884	0.1161	6.95	0.0499
**B**_06_	6.9872	6.95	0.0368	7.014	−0.0264
**B**_07_	6.9245	6.978	−0.0539	6.907	0.0174
**B**_08_ *	6.4078	6.183	0.2249	6.178	0.2301
**B**_09_	6.2328	6.232	0.0007	6.18	0.0533
**B**_10_	6.1637	6.031	0.1328	6.099	0.0648
**B**_11_	6.0814	6.201	−0.1191	6.207	−0.1256
**B**_12_	7.3565	7.362	−0.0054	7.42	−0.063
**B**_13_	7.1739	7.029	0.1451	7.165	0.0092
**B**_14_	6.5467	6.568	−0.0213	6.51	0.0362
**B**_15_	6.4089	6.597	−0.188	6.449	−0.0404
**B**_16_ *	6.1568	6.365	−0.2081	6.297	−0.1406
**B**_17_	6.1278	6.245	−0.1176	6.286	−0.1586
**C**_01_	8.284	7.976	0.308	8.293	−0.0092
**C**_02_	8.1308	8.108	0.0225	8.09	0.0411
**C**_03_ *	7.9586	7.85	0.1088	7.682	0.2764
**C**_04_	7.7959	7.71	0.0861	7.806	−0.0103
**C**_05_	7.4202	7.664	−0.2433	7.519	−0.0989
**C**_06_	7.301	7.119	0.1821	7.177	0.1243
**C**_07_	7.0757	7.135	−0.0597	7.008	0.0677
**C**_08_	7.0269	6.978	0.0491	7.01	0.0171
**C**_09_	6.9469	7.039	−0.0918	7.04	-0.0929

* Molecules belonging to the test set.

### 3.3. Predictive Power of 3D-QSAR Analyses

**Figure 3:**
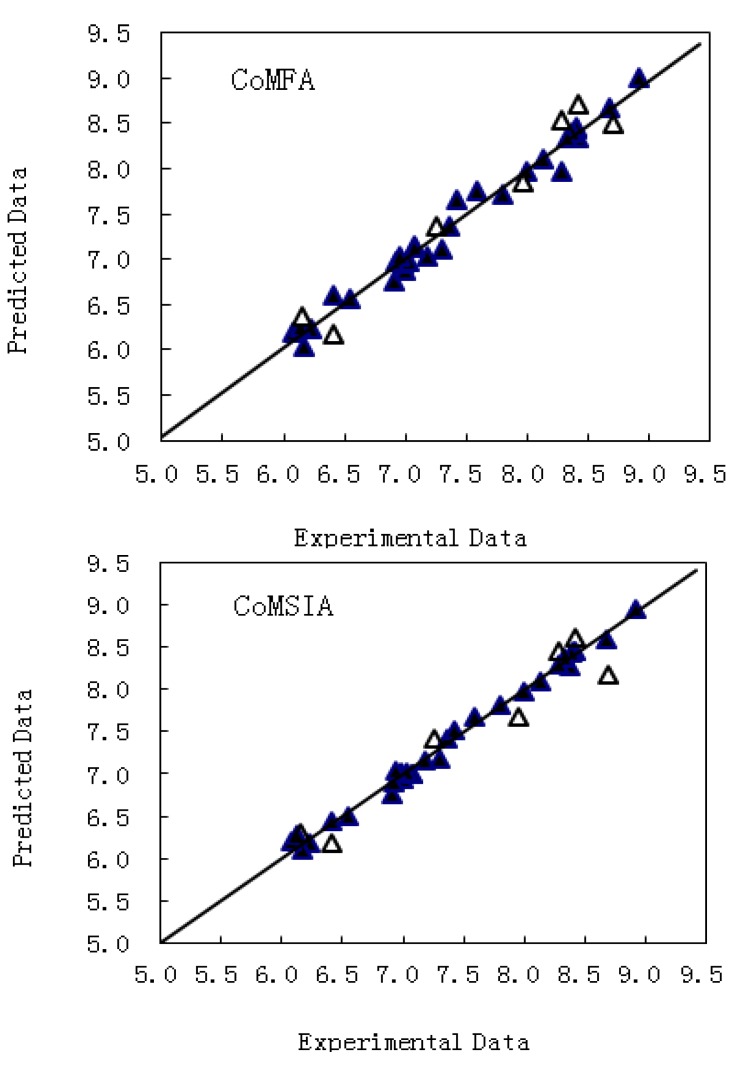
Correlation between the experimental and predicted values of the 3D-QSAR models for the training and test sets. The top figure is the CoMFA model; the bottom one is the CoMSIA model. The training set is shown by the filled triangles; the test set is shown by the empty triangles.

Figure 3 presents the prediction correlation of the CoMFA and CoMSIA models. Most of compounds were located on or near the trend line, implying the predicted pIC_50_ values are in good agreement with the experimental data, so the 3D-QSAR of both the CoMFA and CoMSIA models has good predictive value.

### 3.4. Graphical Interpretation of the Fields

**Figure 4:**
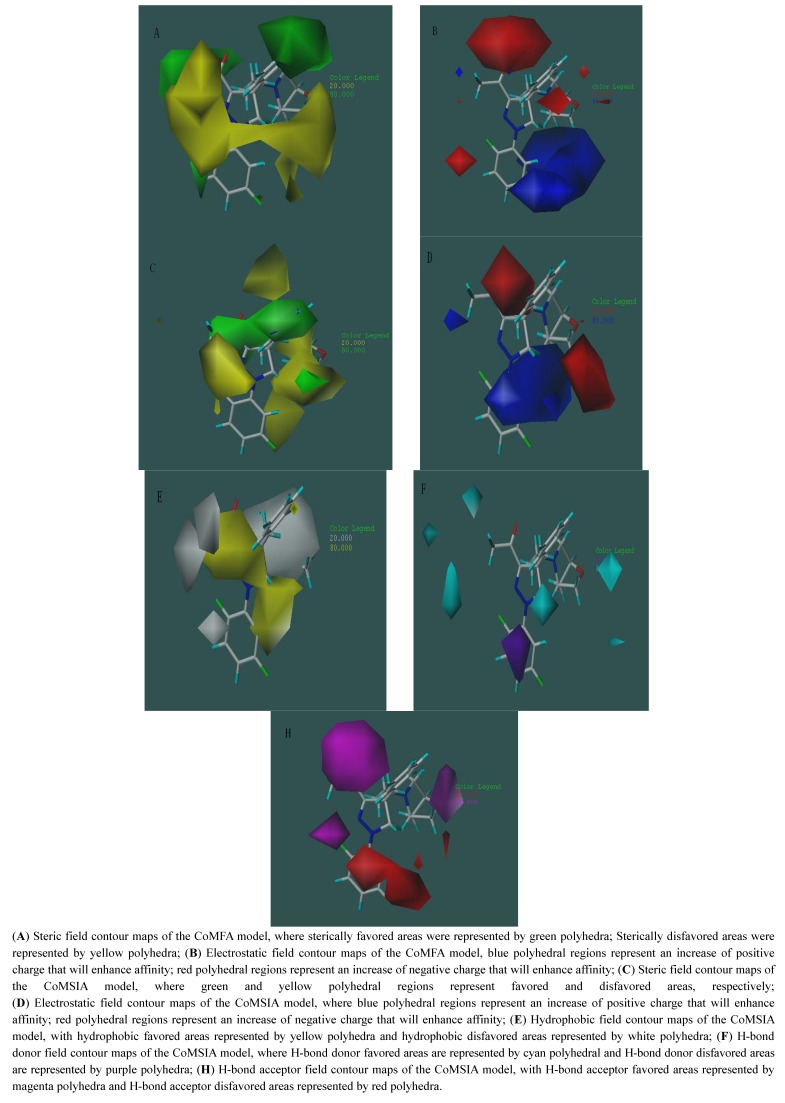
Molecule A_01_ was placed inside of the fields of the CoMFA and CoMSIA models and all the contour cutoff levels were set at 80:20.

The contour maps of different fields contribution of COMFA and COMSIA models are illustrated with inhibitor A_01_ as template (the compound with the highest inhibitive activity of all the Eg5 inhibitors). In the steric field contribution, green areas correspond to regions where steric occupancy with bulky polyhedral groups will increase affinity. Otherwise, yellow polyhedral areas should be sterically avoided. With the CoMSIA steric field map it is easier than with the CoMFA one to illustrate how steric effects affect the activity of inhibitors (Figure 4A,C). The pIC_50_ of A_01_ is 8.92 and that of B_11_ is 6.08 so that we can analyze their steric field contour maps of the CoMSIA model to get more information about the effect of different groups on the activity. In Figure 4C, the difluorobenzene moiety of A_01_ is connected to a pyrazole ring with a nitrogen atom, while in B_11_ it is a carbon atom. Thus, their benzene rings have different spatial locations, the benzene ring’s position in A_01_ is just located in the green region, but the benzene of ring B_11_ is in the yellow region of the steric field, while the R group of B_11_ is also in the yellow area and this reduces the activity. The electrostatic field maps of CoMFA and CoMSIA are shown in Figure 4B,D. Blue polyhedral regions represent an increase of positive charge that will enhance the binding affinity, while red polyhedral regions represent an increase of negative charge that will enhance affinity. In our results, the red areas in the CoMFA are less clear than in the CoMSIA study that displays some little red areas occupied by the ligands that will affect the right design for researchers.

The Figure 4D shows that the negative carboxyl of A_01_ increased affinity in the red region, while B_11_ does not have this effect. In the hydrophobic field contour maps of the CoMSIA model, hydrophobically favored areas were represented by yellow polyhedral and hydrophobically disfavored areas are represented by white polyhedra. Pyrazole rings are surrounded by the yellow polyhedra and also increase the hydrophobic affinity. A large white region on the R groups of A_01_ indicated that hydrophilic properties were important for affinity (see Figure 4E). And high affinity and activity inhibitors would be designed under the analysis of the contour maps in different fields contribution.

In Figure 4F, in the H-bond donor field contour maps of the CoMSIA model, cyan polyhedra represent H-bond donor favored areas; while purple polyhedra represent the H-bond donor disfavored areas. The polyhedron areas in cyan near the pyrazole rings indicate that H on the position will increase binding affinity. The areas in purple near the difluorobenzene moiety of A_01_ proved that the H on that position should decrease binding affinity and H bond acceptors or electron-rich groups should increase binding affinity.

In the H-bond acceptor field maps, H-bond acceptor favored areas are represented by magenta polyhedra while disfavored areas are represented by red polyhedra. In the Figure 4H, the heterocyclic carboxyl oxygen and the fluorine of A_01_ encompassed by the large magenta areas show that a good H-bond acceptor, the difluorobenzene moiety of A_01_, is surrounded by the red polyhedra, indicating that hydrogen in these areas on the ligands represents a low binding affinity.

## 4. Conclusions

Dihydropyrazole and dihydropyrrole derivatives have been described as novel and potent Eg5 inhibitors by Merck Research Laboratories [16,17,18]. These inhibitors have also been proven to have the potential to overcome the multidrug resistance of anticancer drugs, In this work, molecular docking and 3-D QSAR studies were carried out to explore the binding mechanism of dihydropyrazole and dihydropyrrole derivatives to EG5. Good prediction COMFA and COMSIA models were obtained with LOO cross-validation q^2^ and conventional r^2^ values of 0.898, 0.980, and 0.848, 0.992, respectively. The results show that ligands binding in the hydrophobic part of the inhibitor-binding pocket were found to be crucial for potent ligand binding and kinase selectivity. It is thus possible to gain insights into the relationship between the structural information of dihydropyrazole and dihydropyrrole inhibitors and their inhibitory potency, aimed at providing valuable guidance for the design of EG5 inhibitors with high anticancer activity.
